# The rising tide of workplace violence in the healthcare sector: a global scoping review of burden, determinants, and prevention strategies (2021–2025)

**DOI:** 10.3389/fpubh.2026.1851734

**Published:** 2026-05-14

**Authors:** Francesca Simona Fiorino, Caterina Oliveri, Silvia Vivarelli, Concettina Fenga

**Affiliations:** Department of Biomedical and Dental Sciences, Morphological and Functional Imaging, Section of Occupational Medicine, University of Messina, Messina, Italy

**Keywords:** healthcare workers, occupational health, psychosocial risk, violence prevention, workplace violence

## Abstract

**Introduction:**

Workplace violence (WPV) is a pervasive occupational and public health concern affecting healthcare workers (HCWs) globally. This review aims to systematically map recent evidence on WPV, examining its prevalence, associated risk factors, health and occupational consequences, and preventive strategies across different healthcare settings and geographic contexts.

**Methods:**

This scoping review synthesizes evidence from 77 studies conducted across 53 countries and published between 2021 and 2025. Studies reporting data on WPV prevalence, determinants, health consequences, or preventive strategies among HCWs were included, with attention to geographic and socioeconomic variation.

**Results:**

Psychological and verbal violence were the most frequently reported forms of WPV, often affecting over 40–70% of healthcare workers in high-risk environments. Physical and sexual violence, although less prevalent, remained significant, particularly in emergency, psychiatric, and long-term care settings. Nurses, early-career professionals, and women were consistently identified as high-risk groups. Perpetrators included patients, relatives, colleagues, and supervisors. Determinants spanned individual, organizational, and contextual levels, including patient acuity, staffing shortages, weak institutional policies, and socio-political instability. WPV was associated with burnout, anxiety, depression, reduced job satisfaction, turnover, and impaired patient care. Preventive strategies were identified at multiple levels, although evidence for integrated and context-specific approaches remains limited, particularly in low-resource settings.

**Discussion:**

Addressing WPV as a systemic occupational health risk requires coordinated, context-sensitive approaches and rigorous evaluation of interventions to support HCWs, improve reporting, and mitigate adverse outcomes. Future research should prioritize context-sensitive evaluation of interventions, including emerging AI-based approaches, to develop scalable and sustainable prevention.

## 1 Introduction

Workplace violence (WPV) is increasingly recognized as a major occupational and public health concern in healthcare settings, with significant implications for physical safety, mental health, and work ability of healthcare workers (HCWs) (1). Compared with other occupational groups, HCWs are excessively exposed to WPV, which contributes substantially to the global burden of occupational injuries and psychosocial risks (2). WPV has been associated with adverse psychological outcomes, reduced job satisfaction, absenteeism, and workforce turnover, with downstream effects on patient safety and health system performance (3, 4).

According to the World Health Organization (WHO) and International Labour Organization (ILO), WPV includes physical, psychological, and sexual violence or harassment in work-related contexts (5). In healthcare, it comprises physical assault, verbal abuse, bullying, and sexual harassment, perpetrated by patients, relatives, the public, or coworkers (6). Risk is amplified by frequent interactions, emotional demands, time pressure, and shift work (7). High-risk settings embrace emergency departments, psychiatric services, intensive care units, and long-term care facilities (8). Psychological violence, particularly verbal abuse, is the most prevalent form, disproportionately affecting women and frontline staff (e.g., nurses, residents, students) (9).

Despite its prevalence, psychological violence remains underrecognized in occupational surveillance systems (10). Repeated exposure is associated with chronic stress, reduced work ability, and intention to leave, underscoring its relevance for preventive medicine and workforce sustainability (11). These risks were exacerbated during COVID-19 pandemic, particularly in overstretched systems, with increased incidents and persistent psychological effects among HCWs (12).

The epidemiology of WPV varies across settings and regions. Structural determinants (including national income, health system capacity, governance, and socio-political stability) may influence both occurrence and reporting (13). Both low-resource and high-income settings report high WPV prevalence, suggesting that structural factors influence not only occurrence but also detection and reporting (14–16).

WPV is considered preventable through multilevel interventions targeting organizational, environmental, and structural determinants (17). Primary prevention includes risk assessment, staffing, environmental safety, training, reporting systems, and policies, while secondary and tertiary approaches focus on early identification, psychosocial support, and rehabilitation (18).

Although research on WPV in healthcare has expanded, the evidence remains fragmented, particularly regarding prevention effectiveness (19). Heterogeneity in definitions and methods limits comparability, supporting a scoping review to map available evidence and identify gaps (20, 21). Existing reviews often focus on specific populations, settings, or pre-pandemic periods, limiting a comprehensive and up-to-date global perspective (20, 22, 23).

This scoping review synthesizes recent evidence (2021–2025) on WPV among HCWs and trainees, describing prevalence, affected groups, determinants, health consequences, and preventive strategies, with attention to geographical and socio-economic variation.

## 2 Methods

### 2.1 Study design and protocol

This scoping review followed the framework by Arksey and O’Malley and the Joanna Briggs Institute (JBI) guidance (24). Reporting adhered to PRISMA-ScR checklist (25). A protocol was developed *a priori*. Although not registered, the protocol followed established frameworks (21, 26).

### 2.2 Eligibility criteria

Studies were eligible if they: (1) were original empirical studies published in English between January 1, 2021, and December 31, 2025; (2) examined WPV (including harassment, bullying, or sexual violence) among HCWs or trainees in healthcare settings; and (3) reported data on prevalence, determinants, consequences, or prevention. Exclusion criteria were: (1) non-empirical publications (e.g., editorials, commentaries); (2) studies outside healthcare settings; or (3) populations not involving HCWs.

### 2.3 Search strategy

We systematically searched PubMed/MEDLINE, Scopus, and Web of Science for studies published between January 1, 2021, and December 31, 2025 (final update: March 2026). The selected time frame captures post- and late-pandemic evidence, providing an updated synthesis beyond pre-2020 literature. Reference lists were also screened. Search strategies combined controlled vocabulary (e.g., MeSH) and free-text terms for WPV, healthcare personnel, and healthcare settings. Full strategies are provided in Supplementary Tables S1, S2.

### 2.4 Study selection

Titles and abstracts were screened independently by three reviewers. Full texts were assessed for eligibility. The selection process is shown in a PRISMA flow diagram (Supplementary Figure 1). A total of 147 records were identified, and 77 studies were included in the synthesis. Studies published in early 2026 meeting inclusion criteria (*n* = 11) were identified *post hoc* and considered in the Discussion only.

### 2.5 Data charting

Data were extracted using a charting form. Extracted variables included study characteristics, WPV types, and outcomes. Two reviewers independently charted data, with discrepancies resolved through discussion.

### 2.6 Critical appraisal of individual sources of evidence

No formal quality assessment was performed. Methodological limitations of included studies were described narratively where relevant.

### 2.7 Synthesis of results

Given substantial heterogeneity in study designs and outcomes, findings were synthesized narratively. Results were organized by prevalence, types, consequences, and prevention. Geographic distribution was visualized using treemaps (27).

## 3 Results

### 3.1 Population characteristics

As shown in Table 1, the number of studies on WPV in healthcare increased over time, from 7 (9%) in 2021 to 31 (40%) in 2025, indicating growing research attention to this issue. Women predominated in study samples (70%, *n* = 54), whereas male participants were the primary population in 19% (*n* = 15), and gender was unspecified in 10% (*n* = 8). This distribution reflects both the gender composition of the healthcare workforce and the higher reported vulnerability of women to WPV (28).

**Table 1:** Characteristics of included studies: country, sample, population, workplace setting, and year.

Continent	Country	N	Sex (%F/%M)	Population	Workplace setting	Year	Ref.
Africa	Mozambique	140	31/69	HCWs (nurses, physicians, technicians, admin)	Provincial hospitals	2022	(40)
Africa	Ethiopia	534	56/44	Nurses	Public hospitals	2023	(42)
Africa	Ghana	607	82/18	HCWs (nurses, physicians, midwives, lab staff)	Public hospitals	2023	(43)
Africa	Mozambique	260	46/54	HCWs (physicians, nurses, technicians, admin)	Public hospitals	2023	(46)
Africa	South Africa	405	91/9	Nurses (registered/enrolled/auxiliary/community)	Public and private hospitals	2023	(49)
Africa	Ethiopia	640	47/53	Midwives	Public hospitals	2024	(44)
Africa	Ethiopia	1,426	41/59	HCWs (nurses, midwives, physicians, lab/pharmacy staff)	Public hospitals	2024	(41)
Africa	Ethiopia	383	57/43	Nurses	Public hospitals	2024	(48)
Africa	Nigeria	1,218	62/38	HCWs (physicians, nurses, lab staff, CHWs)	Public healthcare facilities	2024	(47)
Africa	Ghana	338	51/49	Nurses	Public hospitals (medical, surgical, pediatric, outpatient depts)	2025	(45)
Asia	Jordan	969	35/65	Physicians	Governmental and university hospitals, private and military centers	2021	(76)
Asia	Taiwan	66	80/20	Senior nursing students	Clinical/community/psychiatric depts	2021	(63)
Asia	Taiwan	102	96/4	Newly employed RNs (<6 months)	Hospitals	2021	(71)
Asia	Republic of Korea	141	60/40	Paramedics	ED	2022	(69)
Asia	Saudi Arabia	849	68/32	Nurses	ED	2022	(51)
Asia	Taiwan	75	97/3	Nurses (full-time)	ED	2022	(52)
Asia	China	1,095	87/13	Nursing students (junior/senior)	Hospitals	2023	(56)
Asia	China	1,567	77/23	HCWs (physicians, nurses, med technicians)	Territorial hospitals	2023	(54)
Asia	China	130	NR	Clinical nurses	Territorial hospitals (ED, ICU, OB-GYN, medical, surgical, outpatient)	2023	(73)
Asia	Israel	122	51/48	HCWs (nurses, physicians, psychologists, social workers)	MH units	2023	(78)
Asia	Turkey	135	60/40	HCWs (physicians, nurses, medical secretaries)	Territorial hospitals	2023	(81)
Asia	China	4,505	70/30	Medical undergraduates	University hospitals	2024	(57)
Asia	China	3,426	73/27	HCWs (physicians, nurses, technicians)	Public hospitals	2024	(53)
Asia	India	164	31/69	Physicians	ED	2024	(65)
Asia	Oman	106	53/47	Nurses	MH, ED and rehab units	2024	(75)
Asia	Saudi Arabia	100	32/68	Physicians	Military and non-military hospital EDs	2024	(55)
Asia	Turkey	945	82/18	Nurses	University hospitals	2024	(79)
Asia	China	116,345	93/7	Nurses	Territorial hospitals	2025	(58)
Asia	China	103	77/23	Nursing undergraduates (3rd year)	University hospitals	2025	(70)
Asia	China	549	87/13	Clinical nurses	Territorial hospitals	2025	(68)
Asia	China	275	94/6	Nurses	Comprehensive hospitals (medical, surgical, pediatric, OB-GYN)	2025	(67)
Asia	China	5,663	92/8	Junior RNs	ED	2025	(59)
Asia	China	63,295	94/6	Early-career nurses	Hospitals	2025	(66)
Asia	China	3,278	93/7	Clinical nurses	Public hospitals	2025	(60)
Asia	China	4,255	NR	HCWs (physicians, nurses/midwives, pharmacists, AHPs, admin)	Public and private hospitals	2025	(61)
Asia	Jordan	431	55/45	Nurses	Government peripheral hospitals (ED, ICU, med-surg units)	2025	(77)
Asia	Taiwan	280	88/12	Nurses	Hospitals (ICU, ED, wards, psychiatry, outpatient)	2025	(72)
Asia	Taiwan	119	89/11	Nurses	ED	2025	(74)
Asia	Taiwan	101	82/18	HCWs (physicians, nurses, pharmacists, therapists, lab & admin)	Hospitals	2025	(64)
Asia	Taiwan	937	84/16	Nursing assistants	LTC facilities	2025	(62)
Asia	Turkey	132	NR	HCWs (physicians, nurses, admin)	Hospitals	2025	(80)
Asia	Turkey	400	83/17	Nurses	Hospitals	2025	(82)
Asia	Turkey	310	37/63	HCWs (nurses, physicians, technicians, midwives, admin)	State hospitals	2025	(83)
Asia	Turkey and Palestine	377	37/63	HCWs (physicians, nurses, admin)	ED	2025	(84)
Europe	Belgium	196	46/54	Physicians	Hospitals (incl. ED)	2021	(95)
Europe	Switzerland	1,128	70/30	Nurses	Psychiatric hospitals	2021	(98)
Europe	Italy	3,659	69/31	Nurses & physicians	Public Hospitals	2022	(86)
Europe	Italy	82	83/17	HCWs (nurses, physicians, other staff)	Territorial Hospitals (pediatric)	2022	(87)
Europe	France	48	58/42	Nurses	ED	2023	(99)
Europe	Multiple EU Countries (Italy, Spain, Cyprus, Greece, Portugal, United Kingdom, Lithuania, Croatia, Germany, Slovenia, Sweden, Albania, Czech Republic, Denmark, Finland, Estonia, France, Ireland, Switzerland, Belgium, Slovakia, Iceland)	28	NR	Nurses	Hospitals	2023	(104)
Europe	United Kingdom	1,434	52/48	Surgeons & surgical trainees	Surgical depts	2023	(96)
Europe	United Kingdom	127	NR	Nurses & physicians	ED	2023	(97)
Europe	Italy	174	55/45	Vascular surgeons	Vascular surgery dept	2024	(91)
Europe	Italy	396	NR	HCWs (physicians, nurses, HC assistants, other staff)	LHA	2024	(88)
Europe	Italy	321	78/22	Nurses	Public health services	2024	(89)
Europe	Spain	2,851	73/27	Physical therapists	Public and private hospitals	2024	(103)
Europe	Spain	120	62/38	HCWs (physicians, nurses, assistants, admin, other staff)	ED	2024	(101)
Europe	Spain	205	NR	HCWs (nurses, physicians, admin)	Pediatric ED	2024	(102)
Europe	Germany	411	77/23	Nurses	LTC facilities, hospitals and outpatient settings	2025	(100)
Europe	Italy	3,259	NR	HCWs (nurses, physicians, MH workers)	Hospitals and LHA	2025	(92)
Europe	Italy	271	70/30	HCWs (nurses, physicians, security)	ED	2025	(93)
Europe	Italy	997	57/43	HCWs (physicians, residents, nurses, HC auxiliaries)	University hospitals	2025	(94)
Europe	Italy	415	67/33	HCWs (physicians, nurses, midwives, admin/technical staff)	University hospitals	2025	(90)
North America	United States	480	59/39	HCWs (clinical & non-clinical staff)	ED	2022	(105)
North America	United States	208	19/81	Orthopedic surgery residents	Orthopedic surgery dept	2024	(111)
North America	United States	58	41/57	HCWs (nurses, physicians, technicians, security)	Teaching hospital ED	2025	(115)
North America	United States	399	100/0	Women physicians	PM&R and orthopedic surgery depts	2025	(114)
North America	United States	262	NR	Nurses	Territorial hospitals	2025	(110)
North America	United States	23	100/0	Cardiologists (female)	Cardiology dept	2025	(113)
North America	United States	222	79/19	Clinicians & nurses	Urban and rural hospitals	2025	(112)
North America	United States	17	88/12	Home care & LTC workers	Home care and LTC settings	2025	(106)
North America	United States and Canada	289	97/3	Residents/fellows/physicians	Ophthalmology dept	2025	(109)
North & South America	Latin America (Mexico, Chile, Argentina, Perù)	254	88/12	Oncologists	Territorial hospitals	2025	(107)
South America	Colombia	302	42/58	General surgery residents	General surgery dept	2023	(116)
Oceania	Australia	36	64/36	Nurses	MH units	2021	(117)
Multiple Continents	Multiple countries (not specified)	5,931	23/77	Cardiologists	Public and private health sector	2021	(118)
Multiple Continents	Multiple countries (Italy, Spain, Cyprus, Norway, Greece, Portugal, United Kingdom, Netherlands, Poland, Lithuania, North Macedonia, Romania, Bosnia and Herzegovina, Croatia, Germany, Slovenia, Sweden, Turkey, Australia, Israel, Lebanon, United Arab Emirates, Saudi Arabia)	483	NR	Nurses	Renal units	2022	(119)

HCWs, healthcare workers; RNs, registered nurses; AHPs, allied health professionals; ED, emergency department; ICU, intensive care unit; OB-GYN, obstetrics and gynecology; MH, mental health; LTC, long-term care; LHA, local health authority; PM & R, physical medicine & rehabilitation; med-surg, medical-surgical; depts, departments; NR, not registered; M, males; F, females.

Regarding professional groups, studies most frequently included mixed HCW populations (35%, *n* = 27) and nurses alone (36%, *n* = 28). Physicians accounted for 10% (*n* = 8), while students, trainees, residents, and fellows represented 9% (*n* = 7). Other groups were marginally represented (≤3%) (29). Most studies were conducted in general or multi-specialty hospitals (51%, *n* = 39), followed by emergency departments (EDs, 18%, *n* = 14) and public/provincial hospitals (14%, *n* = 11). EDs were among the most frequently examined high-risk settings (30).

### 3.2 Geographic and socioeconomic distribution

**Figure 1:**
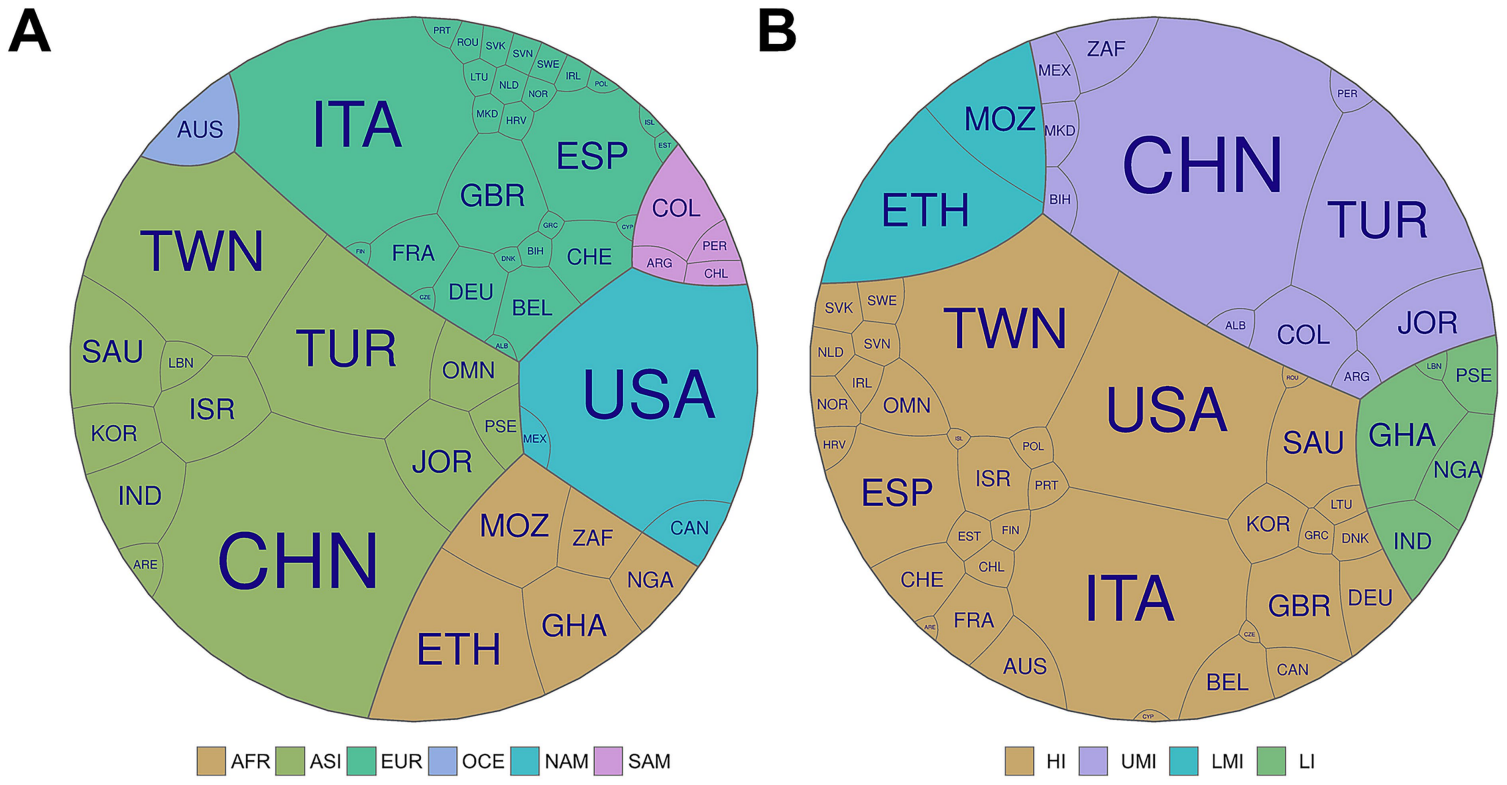
Voronoi diagrams of included countries grouped by continent **(A)** and by income level **(B)**. Each polygon represents a country included in the review. In panel **(A)**, colors indicate continental affiliation: Africa (AFR), Asia (ASI), Europe (EUR), North America (NAM), South America (SAM), and Oceania (OCE). In panel **(B)**, colors represent World Bank income classifications: high income (HI), upper-middle income (UMI), lower-middle income (LMI), and low income (LI). ETH, Ethiopia; MOZ, Mozambique; GHA, Ghana; NGA, Nigeria; ZAF, South Africa; ARE, United Arab Emirates; ISR, Israel; KOR, South Korea; OMN, Oman; SAU, Saudi Arabia; TWN, Taiwan; IND, India; LBN, Lebanon; PSE, Palestine; CHN, China; JOR, Jordan; TUR, Turkey; BEL, Belgium; CHE, Switzerland; CYP, Cyprus; CZE, Czech Republic; DEU, Germany; DNK, Denmark; ESP, Spain; EST, Estonia; FIN, Finland; FRA, France; GBR, United Kingdom; GRC, Greece; HRV, Croatia; IRL, Ireland; ISL, Iceland; ITA, Italy; LTU, Lithuania; NLD, Netherlands; NOR, Norway; POL, Poland; PRT, Portugal; ROU, Romania; SVK, Slovakia; SVN, Slovenia; SWE, Sweden; ALB, Albania; BIH, Bosnia and Herzegovina; MKD, North Macedonia; CAN, Canada; USA, United States; MEX, Mexico; CHL, Chile; ARG, Argentina; COL, Colombia; PER, Peru; AUS, Australia.

The 77 included studies covered 53 countries across six continents (Table 2). Most originated from Asia (45%) and Europe (26%), followed by Africa (13%), North America (12%), Latin America (2%), and Oceania (1%). High- and upper-middle-income countries accounted for the majority of publications (87%), whereas lower-middle- and low-income countries represented 8 and 6%, respectively. This imbalance likely reflects disparities in research capacity and publication practices, with well-resourced systems better able to generate and disseminate evidence (Figure 1) (31).

**Table 2:** Country-level distribution and socioeconomic characteristics of included studies.

Continent	Country	% of total publications	Income level	Healthcare system	Geopolitical area	Main language	Current conflicts
AFR	ZAF	1.32%	UMI	MIX	Southern Africa/SADC	English/Afrikaans/Zulu	No
AFR	GHA	2.63%	LMI	NHI	West Africa/ECOWAS	English	No
AFR	NGA	1.32%	LMI	MIX	West Africa/ECOWAS	English	Boko Haram/banditry
AFR	ETH	5.26%	LI	PUB	East Africa/African Union	Amharic	Tigray conflict
AFR	MOZ	2.63%	LI	PUB	Southern Africa/SADC	Portuguese	Cabo Delgado insurgency
ASI	ARE	0.06%	HI	MHI	Middle East/GCC/OPEC	Arabic	No
ASI	ISR	1.37%	HI	NHI	Middle East/Western Asia	Hebrew	Israel-Palestine tensions
ASI	KOR	1.32%	HI	NHI	East Asia & Pacific/OECD	Korean	North Korea tensions
ASI	OMN	1.32%	HI	PUB	Middle East/GCC	Arabic	No
ASI	SAU	2.69%	HI	PUB	Middle East/GCC/G20	Arabic	Yemen conflict
ASI	TWN	9.21%	HI	NHI	East Asia & Pacific/OECD	Mandarin	Cross-strait tensions
ASI	CHN	17.11%	UMI	PUB-SHI	East Asia & Pacific/BRICS	Mandarin	Xinjiang/Hong Kong/Taiwan unrest
ASI	JOR	2.63%	UMI	MIX	Middle East/Western Asia	Arabic	No
ASI	TUR	7.3%	UMI	MIX-SHI	Middle East/NATO/G20	Turkish	Kurdish insurgency
ASI	IND	1.32%	LMI	MIX-GOV	South Asia/BRICS	Hindi/English	Pakistan border tensions
ASI	LBN	0.06%	LMI	MIX	Middle East/Western Asia	Arabic	Lebanon internal instability
ASI	PSE	0.66%	LMI	UN	Middle East/Western Asia	Arabic	Israel-Palestine tensions
EUR	BEL	1.37%	HI	SHI	Western Europe/EU	French/Dutch	No
EUR	CHE	1.37%	HI	MHI	Western Europe/EFTA	German/French/Italian	No
EUR	CYP	0.12%	HI	NHI	Southern Europe/EU	Greek/Turkish	No
EUR	CZE	0.06%	HI	SHI	Central Europe/EU	Czech	No
EUR	DEU	1.43%	HI	SHI	Western Europe/EU/G7	German	No
EUR	DNK	0.06%	HI	NHS	Northern Europe/EU/NATO	Danish	No
EUR	ESP	4.07%	HI	NHS	Western Europe/EU/OECD	Spanish	No
EUR	EST	0.06%	HI	NHS	Northern Europe/EU/NATO	Estonian	No
EUR	FIN	0.06%	HI	NHS	Northern Europe/EU/NATO	Finnish	No
EUR	FRA	1.37%	HI	SHI	Western Europe/EU/G7	French	No
EUR	GBR	2.75%	HI	NHS	Western Europe/Europe	English	No
EUR	GRC	0.12%	HI	NHS	Southern Europe/EU	Greek	No
EUR	HRV	0.12%	HI	SHI	Southern Europe/EU	Croatian	No
EUR	IRL	0.06%	HI	MIX-NHS	Western Europe/EU	English	No
EUR	ISL	0.06%	HI	NHS	Northern Europe/EFTA/NATO	Icelandic	No
EUR	ITA	11.96%	HI	NHS	Western Europe/EU/G7	Italian	No
EUR	LTU	0.12%	HI	NHS	Northern Europe/EU/NATO	Lithuanian	No
EUR	NLD	0.06%	HI	SHI	Western Europe/EU/NATO	Dutch	No
EUR	NOR	0.06%	HI	NHS	Northern Europe/EFTA/NATO	Norwegian	No
EUR	POL	0.06%	HI	SHI	Central Europe/EU/NATO	Polish	No
EUR	PRT	0.12%	HI	NHS	Western Europe/EU/NATO	Portuguese	No
EUR	ROU	0.06%	HI	SHI	Eastern Europe/EU	Romanian	No
EUR	SVK	0.06%	HI	SHI	Central Europe/EU/NATO	Slovak	No
EUR	SVN	0.12%	HI	SHI	Southern Europe/EU	Slovene	No
EUR	SWE	0.12%	HI	NHS	Northern Europe/EU/NATO	Swedish	No
EUR	ALB	0.06%	UMI	MIX	Balkans/NATO	Albanian	No
EUR	BIH	0.06%	UMI	SHI	Balkans/Europe	Bosnian/Serbian/Croatian	No
EUR	MKD	0.06%	UMI	SHI	Balkans/NATO	Macedonian	No
NAM	CAN	0.66%	HI	NHS	North America/OECD/G7	English/French	No
NAM	USA	11.19%	HI	EMP	North America/OECD/G7	English	No
NAM	MEX	0.33%	UMI	MIX-SHI	North America/Latin America	Spanish	Drug-related violence
SAM	CHL	0.33%	HI	MIX-SHI	Latin America/UNASUR	Spanish	Mapuche conflict/social unrest
SAM	ARG	0.33%	UMI	MIX-SHI	Latin America/UNASUR	Spanish	Protests/social tensions/economic instability
SAM	COL	1.32%	UMI	MIX-SHI	Latin America/UNASUR	Spanish	FARC residual violence
SAM	PER	0.33%	UMI	MIX-SHI	Latin America/UNASUR	Spanish	Peru internal conflicts/social tensions
OCE	AUS	1.37%	HI	MIX-MED	Oceania / OECD / G20	English	No

LI = low income; LMI = lower middle income; UMI = upper middle income; HI = high income; MIX = mixed public-private; NHI = national health insurance; PUB = publicly funded; MHI = mandatory/mixed; PUB-SHI = Public + SHI; SHI = social health insurance; SHI-MIX = SHI/public-private; MIX-GOV = Mixed/Government; UN = Public/UN; MIX-NHS = Mixed/NHS; MIX-SHI = Mixed/SHI; MIX-MED = Mixed/Medicare (Australia); EMP = Employer-based/Mixed; NHS = national health service; SADC = Southern African Development Community; ECOWAS = Economic Community of West African States; GCC = Gulf Cooperation Council; OECD = Organisation for Economic Co-operation and Development; BRICS = Brazil, Russia, India, China, South Africa; NATO = North Atlantic Treaty Organization; UNASUR = Union of South American Nations; main language = official language(s). Percentages were calculated using fractional counting for multicountry studies (1/n). For one study, the countries involved were not specified; therefore, fractional allocation could not be performed.

Health system structures varied across countries. Such variation is relevant, as resource constraints, workforce shortages, as well as weaker occupational protections, are more common in lower-income and mixed systems and may increase vulnerability to WPV (32, 33).

Approximately one-third of countries (32%) were affected by conflict or socio-political instability. These contexts may increase exposure while limiting reporting (34–36). Results from Africa and Latin America suggests that conflict, economic instability, and social unrest may simultaneously increase risk and reduce documentation of WPV (37, 38). Overall, the geographic distribution highlights important gaps in evidence. The predominance of high-income settings limits generalizability to lower-resource contexts (6, 39).

### 3.3 Global burden and patterns of workplace violence in healthcare settings

#### 3.3.1 Africa

Ten cross-sectional studies (2022–2025) from Ethiopia, Ghana, Mozambique, South Africa, and Nigeria examined WPV among HCWs, primarily in hospital settings and predominantly involving female participants (Table 3) (40–43).

**Table 3:** Global workplace violence in healthcare: features of the included studies.

Continent	Country	Violence type	Source	Consequences of violence	Study design	Assessment tools	Intervention	Ref.
AFR	ETH	PSY (verbal abuse, bullying); PHY; SEX	EXT; INT	W: Psychological distress, burnout, low job satisfaction, poor performance, substance use; O: reduced productivity, financial/occupational loss	Cross-sectional	WPV questionnaire (adapted from ILO/ICN/WHO/PSI)	None	(42)
AFR	ETH	PSY (verbal abuse); PHY	EXT; INT	W: Burnout, emotional exhaustion, decreased job satisfaction, negative physical and psychological effects, higher intention to leave; O: reduced quality of maternity care, potential medical errors; P: increased medical errors	Cross-sectional	Copenhagen Burnout Inventory; Structured questionnaire for sociodemographic, work-related factors, and violence	None	(44)
AFR	ETH	PSY (verbal violence); PHY	EXT; INT	W: Depressive symptoms, anxiety, occupational stress	Cross-sectional	Perceived Occupational Stress Scale; Occupational Depression Inventory; Job Anxiety Scale; Insomnia Severity Index; Life Events questionnaire	None	(41)
AFR	ETH	PSY (verbal abuse); PHY; SEX	EXT; INT	W: Job dissatisfaction, job stress	Cross-sectional	WPV questionnaire (adapted from ILO/ICN/WHO/PSI)	None	(48)
AFR	GHA	PSY (verbal abuse); PHY (assault); SEX (harassment)	EXT; INT	W: Hypervigilance, disturbing memories, avoidance; O: absenteeism, stress, turnover	Cross-sectional	WPV questionnaire adapted from multiple instruments	None	(43)
AFR	GHA	PSY (verbal aggression, bullying); PHY (attacks); SEX (assault, rape)	INT	W: Burnout, imposter syndrome	Cross-sectional	Structured questionnaire adapted from Negative Acts Questionnaire-Revised (NAQ-R); General Health Questionnaire-15 (GHQ-15)	None	(45)
AFR	MOZ	PSY (verbal threats, bullying/mobbing); PHY (assault); SEX (advances)	EXT; INT	W: Emotional distress, anxiety, burnout	Cross-sectional	Personalized WPV questionnaire (7 sections)	None	(40)
AFR	MOZ	PSY (verbal aggression, bullying, mobbing); PHY (hitting); SEX (harassment, unwanted advances)	EXT; INT	W: Burnout, poor mental health	Cross-sectional	WPV questionnaire (adapted from ILO/ICN/WHO/PSI)	None	(46)
AFR	NIG	PSY (verbal abuse, threats, bullying, harassment, emotional blackmail, sarcasm, gossiping, false allegations); PHY (pushing, slapping, grabbing, jacking, tearing clothes, hitting/kicking, biting/pinching, jerking shirt, throwing objects, stabbing, shooting)	EXT; INT	W: Lack of job satisfaction, reduced productivity, loss of confidence, PTSD, minor injuries, time off work	Cross-sectional	Standardized WPV questionnaire	None	(47)
AFR	SOU	PSY (verbal abuse, incivility, bullying, mobbing, harassment, threats); PHY (grabbing, punching, chasing, pushing, pinching, scratching, kicking, biting, hair pulling, stabbing, urinated/defecated on)	INT	W: Job dissatisfaction, career dissatisfaction, intention to leave, poor physical and mental health	Cross-sectional	Negative Acts Questionnaire-Revised (NAQ-R); RN4CAST single-item measures; Patient-Reported Outcomes Measurement Information System 10-item Global Health Short Form (PROMIS-10)	None	(49)
ASI	CHI	PSY	EXT; INT	W: Reduced professional commitment, emotional distress, anxiety	Cross-sectional	WPV questionnaire (Chinese version); WHO WPV Scale; Professional Commitment of Nurses Scale	None	(56)
ASI	CHI	PSY (verbal violence)	EXT	W: Emotional exhaustion, reduced job satisfaction, lower work engagement	Cross-sectional	WPV questionnaire; Differential Emotions Scale; Maslach Burnout Inventory; Minnesota Satisfaction Questionnaire	None	(54)
ASI	CHI	PSY (verbal aggression, verbal threats); PHY (aggression); SEX (verbal and PHY harassment)	EXT	W: Chest tightness, headaches, minor injuries, fear, anxiety, depression, insomnia, decreased job satisfaction, lower efficiency; O: higher turnover intention, absenteeism	Quasi experimental	Revised WPV questionnaire; WPV severity grading scale; Hospital WPV coping resources scale	Development of WPV prevention strategy based on situational prevention theory and 11 high-risk situational elements, delivered with classroom teaching and simulations over 10 months. WPV incidence decreased, psychological and physical violence severity decreased, Coping resources increased	(73)
ASI	CHI	PSY; PHY	EXT	W: Worsening mental health, depression, anxiety, fatigue, stress, burnout, decreased life satisfaction	Cross-sectional	Patient Health Questionnaire-9 (PHQ-9, depression); Generalized Anxiety Disorder-7 (GAD-7); Burnout scale; Perceived Stress Scale (PSS); Epworth Sleepiness Scale (ESS); PHQ-15; Alcohol Use Disorders Identification Test - Consumption (AUDIT-C)	None	(57)
ASI	CHI	PSY (verbal violence); PHY	EXT	W: Burnout, psychological strain, stress reactions, reduced patience, fatigue; O: potential impact on service quality and safety	Cross-sectional	WPV questionnaire	None	(53)
ASI	CHI	PSY (emotional abuse); PHY (assault); SEX (harassment)	EXT	W: Depressive symptoms, stress, burnout; O: turnover intentions	Cross-sectional	WPV scale (Chinese version); PHQ-9; GAD-7; PSS-4; single-item Burnout question; single-item Turnover Intention question	None	(58)
ASI	CHI	PSY (harassment, intimidation, disruptive/threatening actions); PHY (assault);	EXT	W: Physical harm, decreased confidence, depression, anxiety, burnout	Randomized controlled	Objective Structured Clinical Examination (OSCE, 3 stations); WPV Management Ability Assessment Questionnaire; Knowledge Test; Confidence in Coping with Patient Aggression Instrument (CCPA)	Theme game-based learning intervention covering risk factor identification, hazardous item management, pre-violence indicators, personnel division, verbal de-escalation, and protective restraint application. Observed results: Significant improvement in violence risk assessment and response skills (OSCE), workplace violence management ability, and self-confidence	(70)
ASI	CHI	PSY (verbal abuse, threats); PHY (assault); SEX (harassment)	EXT	W: Burnout including emotional exhaustion, depersonalization, reduced personal accomplishment, resource depletion, emotional labor, impaired resilience	Cross-sectional	WPV Scale (Chinese, 5 items); Connor-Davidson Resilience Scale (CD-RISC, 25 items); Emotional Labor Scale (ELS, 14 items); Maslach Burnout Inventory - Human Services Survey (MBI-HSS, 22 items); Perceived Organizational Support scale (POS, 9 items)	None	(68)
ASI	CHI	PSY (insults, humiliation, offensive language); PHY (attacks, non-consensual contact);	EXT	W: Increased stress, burnout, lowered job satisfaction	Cross-sectional	Chinese Maslach Burnout Inventory - General Survey (CMBI-GS); PHQ-2; Chinese Pittsburgh Sleep Quality Index (PSQI); WPV Scale (WVS); demographic and occupational questions	None	(67)
ASI	CHI	PSY (emotional abuse, threats); PHY (hitting, grabbing); SEX	EXT	W: Increased risk of mental distress, anxiety, low professional identity, affecting work performance and resilience	Cross-sectional	WPV Scale (Chinese version)	None	(59)
ASI	CHI	PSY (emotional abuse); PHY (assault, threatening intimidation); SEX (verbal and PHY SEX harassment)	EXT; INT	W: Increased risk of depression, anxiety, irritability, fatigue, sleep disturbance, worthlessness, anticipatory fear	Longitudinal prospective	PHQ-9; GAD-7; 5-item WPV Scale	None	(66)
ASI	CHI	PSY (emotional abuse); PHY (hitting, pushing, grabbing); SEX (PHY)	EXT	W: Physical harm, burnout, reduced job satisfaction, lower work performance; O: higher turnover intention, increased job stress	Cross-sectional	WPV Frequency Measurement Scale; Caring Behaviors Inventory; Ten-Item Personality Inventory	None	(60)
ASI	CHI	PSY (emotional abuse, verbal threats); PHY; SEX	EXT	W: Anxiety, depression, PTSD, sleep disturbance, psychological distress	Cross-sectional	WPV Scale (Chinese version)	None	(61)
ASI	IND	PSY; PHY (abuse); SEX	EXT	W: Burnout, reduced job satisfaction	Cross-sectional	Survey on demographics, residency issues, administrative tasks, verbal abuse, and solutions (36 items)	None	(65)
ASI	KOR	PSY (verbal abuse); PHY (threats);	EXT	W: Emotional exhaustion, depersonalization, high burnout; O: reduced performance, satisfaction, productivity	Cross-sectional	Assault Response Questionnaire; Maslach Burnout Inventory	None	(69)
ASI	TAI	SEX (harassment verbal, PHY, visual)	EXT	W: Fear, anger, helplessness, depression, lowered self-concept; reluctance to continue nursing; O: attrition;	Randomized controlled	Sexual Harassment Prevention Concept Scale; Coping Behavior and Prevention Subscale; Instructional Materials Motivation Survey (IMMS)	Interactive multimedia e-book improved knowledge and skills related to sexual harassment prevention compared to traditional methods. In the posttest, the e-book group scored significantly higher in the sexual harassment prevention knowledge and sexual harassment prevention strategy subscales than the control group, but not in the coping behavior subscale.	(63)
ASI	TAI	SEX (harassment verbal, visual, gender/power-related)	EXT	W: Anger, self-blame, fear, depression, anxiety; decreased mental and physical well-being; O: reduced performance, higher risk of errors, potential turnover; P: compromised patient safety	Randomized controlled	Sexual Harassment Prevention Concept Scale; Coping Behavior Scale for Verbal and Nonverbal Sexual Harassment; Sexual Harassment Management and Prevention Strategies Scale; Instructional Materials Motivation Survey	Interactive e-book (ARCS model) with definitions, types, myths, scenarios, coping/prevention strategies, and interactive features. Results: increased knowledge, coping behaviors, prevention strategies, learning motivation.	(71)
ASI	TAI	PSY (verbal aggression/abuse); PHY (aggression)	EXT	W: Physical injury, psychological distress, occupational stress, emotional exhaustion, reduced job satisfaction, decreased coping confidence; O: impaired goal commitment, potential medical errors	Cluster-randomized controlled	Goal Commitment Scale; Objective Structured Clinical Examination - Nursing (OCSE-N); Attitudes Toward Aggression in Emergency Department; Confidence in Managing Aggressive Behavior; Attitudes Toward Aggression Behavior Questionnaire (ATABQ)	WPV-PMTP intervention group coped better with violent situations, more confident in managing patient/relative aggression, improved attitudes toward violence, increased perception of support from management.	(52)
ASI	TAI	PSY (verbal aggression, threats); PHY (assault, hitting, kicking)	EXT	W: Psychological distress (anxiety, depression, stress), reduced performance, lower professional well-being; O: higher burnout, intention to leave; P: compromised patient care	Longitudinal quasi-experimental	General Self-Efficacy Scale; Perception of Aggression Scale; Management of Aggression and Violence Attitude Scale	Brazilian jiu-jitsu training intervention group exhibited significantly greater improvements in self-efficacy and workplace violence perceptions	(72)
ASI	TAI	PSY (verbal abuse, threats); PHY	EXT; INT	W: Psychological stress, reduced coping confidence, impaired communication; O: workplace safety concerns	Randomized controlled	Perception of Aggression Scale for Emergency Department (POAS-ED); Management of Aggression and Violence Attitude Scale (MAVAS-ED); Communication Competence Scale (CCS); Confidence in Coping with Patient Aggression Scale (CCPA); Work Stress Coping Strategies Scale (problem- and emotion-oriented subscales)	120-min Debriefing for Meaningful Learning-enhanced simulation-based learning session, improved perceptions, attitudes, and coping	(74)
ASI	TAI	PSY (verbal); PHY; SEX	EXT	W: Mental distress, burnout, increased psychotropic use	Cross-sectional	Job Content Questionnaire; Effort-Reward Imbalance; Work-to-Family Conflict; Brief Symptom Rating Scale-5 (BSRS-5); Copenhagen Burnout Inventory	None	(64)
ASI	TAI	PSY (verbal); PHY; SEX (harassment)	EXT; INT	W: Poor mental health, client-related burnout	Cross-sectional	Questionnaire on WPV; BSRS-5; Chinese Copenhagen Burnout Inventory	None	(62)
ASI	ISR	PSY (social shaming, threats, bullying, mobbing)	EXT	W: Burnout, lower performance, anxiety, depression	Cross-sectional	Shirom-Melamed Burnout Measure (SMBM); Professional Functioning Scale; Intention to Leave; Exposure to Social Shaming & Bullying; Perceived Risk of Negative Impact	None	(78)
ASI	JOR	PSY (verbal violence); PHY; SEX (abuse)	EXT; INT	W: Fear, anger, sadness, irritability, difficulty sleeping	Cross-sectional	Custom questionnaire on WPV	None	(76)
ASI	JOR	PSY (verbal violence); PHY;	EXT; INT	W: Injuries, anxiety, fear, frustration, impaired communication, burnout, stress; O: job dissatisfaction, absenteeism, increased turnover; P: reduced quality of care	Cross-sectional	Modified WPV in the Health Sector instrument (adapted from ILO/ICN/WHO/PSI)	None	(77)
ASI	OMA	PSY (verbal abuse, bullying); PHY	EXT	W: Psychological stress, burnout, intention to leave; P: reduced care quality	Cross-sectional	WPV questionnaire (adapted from ILO/ICN/WHO/PSI)	None	(75)
ASI	SAU	PSY (verbal abuse, threats); PHY (kicked, slapped, pushed, repelled, objects thrown, spit on, scratched/beaten, clothes ripped, assaulted with weapon); SEX (harassment)	EXT; INT	W: Emotional distress, anxiety, burnout, reduced job satisfaction, sick leave after incident	Cross-sectional	Custom survey (6 sections: demographics, violence prevalence, types, responses, causes, handling strategies)	None	(51)
ASI	SAU	PSY (verbal violence); PHY	EXT; INT	W: Injuries from assault, stress, frustration, potential burnout; O: underreporting, decreased sense of security	Cross-sectional	Structured WPV questionnaire based on prior studies	None	(55)
ASI	TUR	PSY (verbal); PHY (attempts, PHY contact);	EXT	W: Increased psychological stress, decreased job satisfaction, reduced productivity	Retrospective observational	White Code notification records; sociodemographic and workplace data; judicial process data	None	(81)
ASI	TUR	PSY (verbal); PHY (aggression)	EXT; INT	W: Physical injury, emotional distress, burnout, lower psychological well-being, anxiety, depression, insomnia, PTSD, gastrointestinal symptoms; O: absenteeism, turnover, toxic work environment; P: reduced quality of care	Cross-sectional	Exposure to Violence Scale; Flourishing Scale; Copenhagen Burnout Inventory; Connor-Davidson Resilience Scale	None	(79)
ASI	TUR	PSY (verbal); PHY	EXT	W: Physical and psychological harm; O: disruption of healthcare operations, high rate of female-on-female violence	Retrospective observational	White Code reporting system; hospital information management system	Legal regulation and the White Code system were associated with reduced ICU violence and likely acted as a deterrent	(80)
ASI	TUR	PSY; PHY; SEX (aggression)	EXT; INT	W: Emotional distress, anxiety/depression, sleep issues, injuries, pain; O: reduced performance, burnout, turnover intention, alienation, absenteeism, substance use	Cross-sectional	WPV Scale; Copenhagen Burnout Inventory; Intent to Leave Scale; Job Performance Scale; Work Alienation Scale; demographic & violence-experience questionnaire	None	(82)
ASI	TUR	PSY (verbal violence, yelling, insults, threats); PHY; SEX (harassment)	EXT	W: Emotional exhaustion, depersonalization, burnout, decreased motivation; O: turnover intention, underreporting, low managerial support	Cross-sectional	Professional Attitudes at Occupation Inventory (PAOI); Maslach Burnout Scale (MBS); custom questionnaire: demographics, work environment, exposure to violence, coping, institutional responses	None	(83)
ASI	TUR, PAL	PSY (verbal assault, threats); PHY (pushing, pulling, kicking, hitting, throwing instruments/equipment, use of weapons/sharps); SEX (harassment)	EXT; INT	W: Hopelessness, disappointment, fear, anxiety, guilt, desire for revenge; O: minimizing communication/contact with P, reducing time for care, avoiding risky decisions	Cross-sectional	Custom survey based on ILO/ICN/WHO/PSI WPV questionnaire; sections: demographics, physical/non-physical violence, prevention, reporting, impact on care, intention to leave	White Code and legal protections correlated with higher reporting and some legal actions against aggressors	(84)
EUR	BEL	PSY (verbal aggression, emotional abuse, threats, verbal SEX harassment); SEX (assault)	EXT	W: Burnout, worry/anxiety about violence; O: concerns about quality of care and job satisfaction	Cross-sectional	Custom survey on demographics, professional characteristics, exposure and perception of occupational hazards	None	(95)
EUR	ITA, ESP, CYP, GRC, PRT, GBR, LTU, HRV, DEU, SVN, SWE, ALB, CZE, DNK, FIN, EST, FRA, IRL, CHE, BEL, SVK, ISL	PSY (threats, humiliation); PHY (hitting, kicking, biting, scratching); SEX (harassment/assault, unwelcome SEX attention)	EXT; INT	W: Psychological trauma, stress, burnout, emotional exhaustion, humiliation, low job satisfaction; O: reduced working hours, switching to part-time, increased turnover intention	Cross-sectional	Closed and open-ended questionnaire covering experiences of violence, initiatives, training, policies, and challenges	None	(104)
EUR	FRA	PSY (insults, heckling, provocation); PHY (spitting, pushing, hitting);	EXT; INT	W: Emotional distress, burnout, fear	Prospective observational	Observation grid from Emergency Department staff interviews	None	(99)
EUR	GBR	SEX (harassment jokes, comments, messages, advances, coercion; assault unwanted touching, forced contact; rape)	INT	W: Psychological distress, burnout, depression, self-harm, suicidal ideation, reduced job satisfaction, withdrawal, impaired career progression; O: low trust in institutions, underreporting, normalization, reduced retention; P: decreased safety, poorer team performance, increased risk of harm	Cross-sectional	Qualtrics survey; structured items on harassment, assault, rape, demographics	None	(96)
EUR	GBR	PSY (verbal abuse); PHY; SEX (harassment, abuse)	EXT	W: Stress, anxiety, depression, PTSD, burnout; reduced job satisfaction; O: increased absenteeism, higher staff turnover/attrition; P: potentially compromised care	Longitudinal retrospective	Data via Freedom of Information requests and NHS records	None	(97)
EUR	GER	PSY; PHY	EXT	W: PTSD, anxiety, depression, stress, sleep issues, burnout; injuries, sleep problems; O: absenteeism, turnover, workforce shortages, cognitive failure	Cross-sectional	Hospital Aggression and Behavior Scale - User Version (HABS-UG, violence); Impact of Event Scale-Revised (IES-R, PTSD); demographic questionnaire	None	(100)
EUR	ITA	PSY (verbal aggression, insults, threats, intimidation); PHY (aggression including attacks with weapons); SEX (harassment)	EXT; INT	W: Increased stress, negative psychological impact, reduced well-being, decreased productivity; O: productivity loss, absenteeism, possible resignation/abandonment of work, underreporting of incidents, economic costs (treatment, lost productivity)	Cross-sectional	Italian validated WHO WPV in Health Sector (WVHS) questionnaire	None	(86)
EUR	ITA	PSY (verbal abuse); PHY	EXT; INT	W: Psychological impairment, insufficient general health, risk of burnout, absenteeism	Cross-sectional	Modified Overt Aggression Scale (MOAS); General Health Questionnaire-12 (GHQ-12); Short Form-36 Health Survey (SF-36)	None	(87)
EUR	ITA	SEX (harassment verbal/non-verbal, gender harassment, unwanted attention)	EXT; INT	W: Lower job satisfaction, perceived lack of peer support, impact on personal life, psychological distress, burnout risk; O: reduced career opportunities, lower academic involvement, reduced surgical exposure, gender-based discrimination in hiring/promotion, potential reduced recruitment and retention, negative impact on workforce sustainability, possible impact on quality of care	Cross-sectional	Structured non-validated questionnaire (28 items) on gender disparity, job satisfaction, sexual harassment	None	(91)
EUR	ITA	PSY (verbal); PHY (aggression)	EXT; INT	W: Increased stress; O: negative impact on quality of care, staff safety, occupational efficiency	Observational descriptive	Data collected from aggression reporting form	None	(88)
EUR	ITA	PSY (threats, harassment, bullying), PHY (aggression)	EXT; INT	W: Higher occupational stress, fatigue, increased risk of PTSD and burnout, lower professional performance; O: impaired patient care, absenteeism, sickness leave, negative impact on work behavior (conflicts, impaired patient contact, reduced motivation); P: compromised care quality	Perspective panel	Work Ability Index (WAI); Arnetz Violent Incident Form; Demand–Control–Support questionnaire; Effort-Reward Imbalance (ERI)	None	(89)
EUR	ITA	PSY (verbal, threats; cyber persecution); PHY; SEX (harassment)	EXT; INT	W: Physical injuries, psychological distress, decreased job satisfaction, work overload; O: absenteeism, medical errors, decline in organizational performance, underreporting due to fear/stigma, reduced psychological and physical safety; P: compromised care	Cross-sectional	Italian-validated WHO WVHS questionnaire (modified for non-binary gender)	None	(92)
EUR	ITA	PSY (threats, harassment); PHY	EXT	W: Psychological distress, increased workload and stress, job dissatisfaction, post-traumatic stress symptoms; O: communication breakdown among staff, underreporting (especially verbal aggression perceived as “part of the job”); P: potential impact on quality of care	Retrospective observational	Incident reporting forms; Emergency Room access report	None	(93)
EUR	ITA	PSY (verbal violence, bullying); PHY (violence); SEX (harassment)	EXT; INT	W: Increased use of psychotropic drugs, psychotherapy;	Cross-sectional	WHO WVHS survey	None	(94)
EUR	ITA	PSY (verbal aggression, bullying, mobbing); SEX (harassment)	EXT; INT	W: Disturbing memories, avoidance, hypervigilance, fatigue, reduced work ability, isolation	Cross-sectional	WHO Workplace Harassment and Health Survey Questionnaire (WHHSQ); Mini International Personality Item Pool (Mini-IPIP); Brief COPE; Work Ability Index	None	(90)
EUR	SPA	PSY (verbal abuse); PHY; SEX	EXT	W: Psychological harm, impact on health and well-being	Cross-sectional	Custom questionnaire including demographics and violence exposure (career and past 12 months)	None	(103)
EUR	SPA	PSY (verbal aggression, threats, intimidation, harassment); PHY; SEX	EXT; INT	W: PTSD, anxiety, depression, emotional distress (anger, fear, guilt), burnout (emotional exhaustion, cynicism); O: decreased job satisfaction, engagement, productivity; increased absenteeism, errors, reduced quality of care	Cross-sectional	HABS-U (user violence); HABS-CS (coworker/superior violence); GHQ-28; Maslach Burnout Inventory - General Survey (MBI-GS); Utrecht Work Engagement Scale-9 (UWES-9); Minnesota Satisfaction Questionnaire; sociodemographic questionnaire	None	(101)
EUR	SPA	PSY (verbal attacks mild, with gestures); PHY (minor attacks, patient restraint)	EXT	W: Discomfort, anxiety, worry, sadness, fear; minor physical injuries from attacks	Retrospective observational	Voluntary electronic incident reporting system	None	(102)
EUR	SWI	PSY (insults, threats); PHY (biting, spitting, kicking, hitting); SEX	INT	W: Stress, fear, anger, PTSD, reduced empathy, burnout, negative attitude toward patients; serious injuries from physical and sexual assaults, some requiring hospital/long-term treatment; O: decreased job satisfaction, increased absenteeism, elevated staff turnover; P: compromised patient care	Cross-sectional	German version of the Perception of Prevalence of Aggression Scale (POPAS)	None	(98)
NAM	USA	PSY (verbal abuse, threats); PHY (abuse)	EXT	W: Desire to leave profession	Prospective descriptive	Custom survey on verbal/physical abuse, threats, harassment, sexual assault	None	(105)
NAM	USA	PSY (emotional exhaustion, threat); PHY (abuse); SEX (harassment)	EXT; INT	W: Pathological conditions requiring treatment	Cross-sectional	Maslach Burnout Inventory; custom survey on lifestyle satisfaction, belonging, stereotype threat, mistreatment frequency, thoughts of leaving	None	(111)
NAM	USA	PHY (hitting, punching, kicking, spitting, biting, scratching, grabbing, shoving, throwing objects)	EXT	W: Physical harm, safety risk; O: negative impact on staff well-being and care environment	Retrospective observational	Custom data extraction form; hospital incident reporting systems; Electronic Health Records (EHR)	Team-based agitation management protocol with initial reduction in restraint use and physical assaults	(115)
NAM	USA	PSY (sexist comments, gender-based exclusion)	INT	W: Discouragement from entering specialties, negative career experiences, reduced job satisfaction; O: barriers to recruitment, retention, advancement; reinforcement of gender inequality in leadership; reduced inclusivity and equity	Cross-sectional	Custom-designed survey based on Association of American Medical Colleges Matriculating Student Questionnaire (AAMC MSQ)	None	(114)
NAM	USA	PSY (disrespect, harassment, demeaning, bullying, mobbing)	INT	W: Lack of job satisfaction, productivity decline, loss of confidence, PTSD	Cross-sectional	Veterans Affairs All Employee Survey (AES) on job satisfaction, burnout, civility, authenticity	None	(110)
NAM	USA	SEX	INT	W: Burnout, emotional exhaustion, imposter syndrome, decreased self-efficacy, need to conceal authentic self, reduced career satisfaction; O: limited advancement and leadership opportunities, inequitable pay, recognition, promotion, loss of trust in institutional support	Cross-sectional	Semi-structured interview guide audio-recorded	None	(113)
NAM	USA	PSY (bulllying); PHY; SEX	EXT; INT	W: Stress, poor mental health, depression, sleep problems, cognitive impairment; risky drinking; fatigue, low job satisfaction, low engagement, work overload, time pressure; chronic health conditions (e.g., musculoskeletal disorders)	Cross-sectional	National Institute for Occupational Safety and Health Worker Well-Being Questionnaire (NIOSH WellBQ); Qualtrics survey; focus group validation	None	(112)
NAM	USA	PSY (verbal abuse, bullying); PHY	EXT; INT	W: Increased burnout, higher work-related rumination, increased work–family conflict, lower emotional well-being, lower job and life satisfaction; O: increased turnover intention, reduced work-life balance, increased absenteeism, staff turnover	Cross-sectional	Work-Family and Family–Work Conflict Scale; Job and Life Satisfaction; Work-related Rumination Questionnaire; SF-36 Emotional Well-being; Oldenburg Burnout Inventory; Turnover Intention Scale; WPV Reporting Questionnaire	None	(106)
NAM	USA, CAN	SEX (harassment, PHY, unwanted attention, coercion, blackmail);	EXT; INT	W: Impaired ability to work, anxiety, discomfort, humiliation, high prevalence of gender discrimination (feeling undervalued, unequal pay, missed promotions/leadership); O: hostile work environment, avoidant behaviors, reduced career opportunities, job change or leaving career	Cross-sectional	Online custom survey: harassment, frequency, reporting, bystander response, gender discrimination	None	(109)
SAM	COL	PSY (bullying, gender inequity); SEX (unwanted attention, coercion)	INT	W: Burnout, depression, PTSD; O: negative impact on learning environment, education quality; P: compromised patient safety	Cross-sectional	Shortened Negative Acts Questionnaire (NAQ, bullying); self-reported sexual harassment survey	None	(116)
NAM & SAM	MEX, CHL, ARG, PER	PSY (verbal abuse, intimidation, exclusion, gender inequity); SEX (harassment)	EXT; INT	W: Reduced career progression, lower likelihood of leadership roles, wage disparities, negative effects on family planning, psychological stress, barriers to professional development	Cross-sectional	Custom questionnaire (27 items) based on European Society for Medical Oncology Women for Oncology (ESMO W4O)	None	(107)
OCE	AUS	PSY (verbal); PHY (aggression)	EXT	W: Threats to physical safety, emotional strain, impact on well-being	Randomized controlled	Electronic Dynamic Appraisal of Situational Aggression + Aggression Prevention Protocol (eDASA+APP); Dynamic Appraisal of Situational Aggression (DASA); Adapted Overt Aggression Scale (OAS); clinical records and seclusion register	eDASA+APP intervention tested: decrease of: aggressive incidents, verbal aggression, PRN medication use, seclusion episodes; increase of: proactive, noncoercive interventions, early intervention by staff	(117)
GLO	Not Specified	PSY (emotional harassment, discrimination); SEX (harassment)	EXT; INT	W: Hopelessness, disappointment, fear, anxiety, guilt, undervaluation, unfair treatment, dissatisfaction with compensation	Cross-sectional	Custom survey on demographics, emotional harassment, discrimination, sexual harassment, professional satisfaction, patient care impact	None	(118)
GLO	ITA, ESP, CYP, NOR, GRC, PRT, GBR, NLD, POL, LTU, MKD, ROU, BIH, HRV, DEU, SVN, SWE, TUR, AUS, ISR, LBN, ARE, SAU	PSY (insults, humiliation, threats); PHY	EXT	W: Emotional distress, anxiety, burnout, reduced job satisfaction	Cross-sectional	EDTNA/ERCA Violence and Aggression Management Questionnaire	None	(119)

Continent: AFR = Africa; ASI = Asia; EUR = Europe; NAM = North America; SAM = South America; OCE = Oceania; GLO = Global. Country codes (ISO-3): ETH = Ethiopia; GHA = Ghana; MOZ = Mozambique; NIG = Nigeria; SOU = South Africa; CHI = China; IND = India; ISR = Israel; JOR = Jordan; OMA = Oman; SAU = Saudi Arabia; KOR = South Korea; TAI = Taiwan; TUR = Turkey; PAL = Palestine; BEL = Belgium; ITA = Italy; ESP/SPA = Spain; CYP = Cyprus; GRC = Greece; PRT = Portugal; GBR = United Kingdom; LTU = Lithuania; HRV = Croatia; DEU/GER = Germany; SVN = Slovenia; SWE = Sweden; ALB = Albania; CZE = Czech Republic; DNK = Denmark; FIN = Finland; EST = Estonia; FRA = France; IRL = Ireland; CHE = Switzerland; SVK = Slovakia; ISL = Iceland; USA = United States; CAN = Canada; COL = Colombia; MEX = Mexico; CHL = Chile; ARG = Argentina; PER = Peru; AUS = Australia. Violence type: PSY = Psychological violence (e.g., verbal abuse, threats, bullying, harassment); PHY = Physical violence (e.g., assault, hitting, pushing); SEX = Sexual violence (e.g., harassment, assault, coercion). Source of violence: EXT = External (patients, relatives, visitors); INT = Internal (colleagues, supervisors, other staff). Consequences of violence: W = Worker-related outcomes; O = Organizational outcomes; P = Patient-related outcomes.

Psychological and verbal violence were consistently the most prevalent forms. Reported prevalence was high across settings, including approximately 50% in Ethiopia and up to 62% in Nigeria, with bullying and verbal aggression also common in Ghana and Mozambique (40–47). Physical violence was less frequent (4–20%) but consistently reported, while sexual violence, although lower, remained non-negligible (e.g., up to 15% in Ghana) (40, 42–45, 47).

Both external (patients, relatives) and internal (colleagues, supervisors) perpetrators contributed to WPV. External sources predominated in several settings, but internal violence, particularly bullying, was substantial, indicating a dual burden linked to both patient interactions and workplace dynamics (42–48).

All studies relied on cross-sectional, self-reported data, with heterogeneous measurement tools (e.g., Copenhagen Burnout Inventory, NAQ-R, GHQ-15, WHO/ILO-based instruments), limiting comparability (40, 42–47).

Exposure to WPV was consistently associated with adverse outcomes, including burnout, psychological distress, anxiety, depression, reduced job satisfaction, and intention to leave (40–47). In South Africa, exposure was associated with moderate-to-large declines in physical and mental health and a two- to three-fold increase in adverse psychological outcomes among nurses (49).

Underreporting was common, driven by limited awareness of reporting systems and organizational barriers (40, 43, 45–47). Across studies, prevention strategies, such as zero-tolerance policies, reporting systems, staff training, and organizational support, were proposed; but none were formally evaluated. Recommendations emphasized addressing internal workplace dynamics, strengthening reporting mechanisms, and implementing gender-sensitive and mental health support interventions (40–49).

#### 3.3.2 Asia

A total of 34 studies (2021–2025) examined WPV among HCWs across Western and Eastern Asia (Table 3). Populations were heterogeneous, comprising nurses, physicians, students, and allied health staff across diverse clinical settings, with a predominance of female participants in nursing-focused samples (50–52). Across settings, Psychological and verbal aggression predominated, while physical and sexual violence were less frequent but clinically relevant. Patients and relatives were the main perpetrators, although coworker- and supervisor-related violence was also reported. Underreporting was pervasive, driven by fear, perceived futility, or normalization of aggression (53–55).

##### 3.3.2.1 Eastern Asia (China, South Korea, Taiwan, India)

In China, 44% of HCWs in tertiary hospitals reported verbal violence in the previous year, with higher exposure in emergency settings (54). Nursing students reported 45% psychological WPV during clinical placements, with up to 76% of incidents unreported (56). Other studies in China documented multiple forms of WPV, associated with depression, anxiety, fatigue, burnout, and reduced job satisfaction (53, 57–61).

Findings from Taiwan and China were consistent across professions and career stages. Emergency nurses reported 46% non-physical and 19% physical violence, while long-term care assistants experienced verbal (26%), physical (16%), psychological (15%), and sexual (14%) violence (52, 62). Sexual harassment, although less frequent, affected 26% of Taiwanese nursing students (63). Additionally, WPV exposure was linked to mental distress, burnout, and increased psychotropic use (64). In India, physicians reported verbal hostility (40%), physical violence (20%), and inappropriate sexual or gender-related behaviors (37%) (65). Across Eastern Asia, WPV was consistently associated with burnout, emotional exhaustion, anxiety, depression, and reduced job satisfaction, often independently of workload factors (66–69).

Most studies used validated instruments to assess WPV and related outcomes, including measures of violence exposure, mental health (e.g., PHQ-9, GAD-7), and burnout (e.g., Maslach Burnout Inventory, Copenhagen Burnout Inventory) (52–54, 56–61, 63, 64, 66, 68–72).

Several studies evaluated targeted interventions. In China, a quasi-experimental program reduced WPV incidence and severity while improving coping resources (73). Educational and simulation-based interventions (e.g., de-escalation training) improved violence management and coping skills (52, 70, 74). In Taiwan, e-learning and behavioral training (i.e., Brazilian Ju-Jitsu) enhanced knowledge and self-efficacy. However, these interventions primarily targeted individual-level competencies, with limited organizational integration (63, 71, 72).

##### 3.3.2.2 Western Asia (Saudi Arabia, Israel, Jordan, Turkey, Oman, Palestine)

WPV in Western Asia represents a persistent and complex threat to HCWs, particularly nurses and physicians in emergency, intensive, and psychiatric settings, with patients’ families frequently identified as the main perpetrators (51, 55).

High prevalence was reported across countries. In Saudi Arabia, 74% of emergency nurses experienced WPV, predominantly verbal abuse, alongside threats, physical assault, and sexual harassment; over half of incidents were unreported and associated with emotional distress, burnout, and reduced job satisfaction (51, 55, 75). In Jordan, 76% of emergency physicians reported WPV, mainly verbal, with low reporting rates and widespread perceptions that reporting was ineffective. Among nurses, 65% reported moderate-to-high exposure, with individual traits influencing vulnerability (76, 77).

In Oman, WPV affected up to 91% of psychiatric nurses, with high levels of verbal and physical violence, as well as bullying and racial harassment. Despite high awareness of reporting procedures, 63% of incidents were not investigated or reported. Exposure was associated with psychological stress, burnout, intention to leave, and reduced quality of care (75).

In Israel, HCWs in mental health settings reported frequent social shaming, bullying, and online harassment, strongly associated with burnout, impaired professional functioning, intention to leave (78). In Turkey, verbal violence predominated (61–73%), particularly in emergency and outpatient settings, contributing to emotional distress, burnout, and reduced quality of care; the “White Code” reporting system improved reporting and reduced violence in some settings (79–83). In Palestine, WPV affected 85% of emergency department staff, largely driven by patients’ families, with low reporting rates (20%) and significant psychological and occupational consequences (84).

Across studies, WPV was consistently associated with emotional exhaustion, anxiety/depression, reduced job satisfaction, absenteeism, and turnover, with early-career HCWs particularly vulnerable (54, 57, 67). Underreporting remained prevalent due to fear, perceived futility, normalization of aggression, and unclear procedures, suggesting that the true burden is likely underestimated (51, 55, 56).

Most studies used validated self-report instruments (e.g., ILO/ICN/WHO/PSI tools, PHQ-9, GAD-7, Maslach and Copenhagen Burnout Inventories) and were cross-sectional, limiting causal inference and generalizability (57, 66, 68, 69, 73).

Recommended prevention strategies included zero-tolerance policies, accessible and anonymous reporting systems, de-escalation training, improved staffing and patient flow, environmental safety measures, and post-incident psychological support. However, evidence remains limited by study design and the lack of rigorously evaluated, multilevel interventions addressing structural, organizational, and individual determinants of WPV (52, 54, 55, 75).

#### 3.3.3 Europe

A total of 19 European studies (2021–2025) demonstrate high WPV prevalence, persistent underreporting, and marked gender disparities (Table 3). HCWs are predominantly female, particularly nurses, and WPV is most frequently reported in EDs, surgical and pediatric wards, psychiatric hospitals, and long-term care facilities. In these high-income, largely non-conflict countries, WPV risk is primarily linked to occupational, organizational, and cultural factors rather than civil unrest (85–94). WPV encompasses psychological aggression, physical violence, and sexual harassment or assault. Both external sources, such as patients and relatives, and internal sources, including colleagues and supervisors, contribute to risk, complicating reporting and mitigation (95–97).

Country-level studies illustrate the heterogeneity of WPV. In Belgium, predominantly female physicians in hospitals and EDs reported psychological violence and occasional sexual assault, associated with burnout, anxiety, and concerns about quality of care and job satisfaction, with external perpetrators predominating; data were collected via custom cross-sectional surveys, limiting cross-country comparability (95). Psychiatric nurses in Switzerland reported verbal insults, threats, physical aggression, and sexual harassment, with some incidents requiring hospitalization (73% verbal violence, 28% physical violence, 39% verbal sexual, 14% physical sexual), resulting in stress, fear, anger, PTSD, reduced empathy, burnout, lower job satisfaction, increased absenteeism, and compromised patient care; use of the validated POPAS scale allowed robust prevalence estimation (98).

In France, ED nurses experienced insults, heckling, provocation, and minor physical aggression, leading to emotional distress, fear, and burnout, with data collected via prospective observation grids providing qualitative insights (99). In the United Kingdom, surgeons, trainees, and ED staff experienced severe sexual violence, primarily from internal sources, with reporting low at 42%; women were disproportionately affected, and consequences included distress, burnout, depression, self-harm, suicidal ideation, reduced job satisfaction, impaired career progression, and organizational outcomes such as low institutional trust, staff turnover, compromised patient safety (96, 97). In Germany, nurses across hospitals, long-term care, and outpatient settings reported high prevalence of predominantly external WPV (94% non-physical, 76% physical), with PTSD, anxiety, depression, stress, sleep disturbances, burnout, absenteeism, workforce shortages, and compromised patient care; standardized instruments such as HABS-UG and IES-R enabled strong exposure-outcome linkages (100).

In Italy, multiple studies across healthcare settings documented verbal aggression 47–83%, physical violence 10%, internal abuse 11–32%. Psychological consequences included stress, PTSD, burnout, hypervigilance, disturbing memories, fatigue, decreased work ability, sexual harassment-related distress, increased psychotropic use, psychotherapy, and perceived lack of peer support. Occupational consequences involved absenteeism, reduced productivity, resignation, underreporting, communication breakdown, and limited career opportunities; while organizational outcomes comprised medical errors and compromised patient safety (86–94).

In Spain, healthcare professionals reported psychological, physical, and sexual violence from internal and external sources, with lifetime prevalence of sexual violence at 48% and psychological abuse at 43%; outcomes included PTSD, anxiety, depression, emotional distress, burnout, reduced engagement, absenteeism, and minor physical injuries, evaluated via multi-dimensional tools including HABS-U/CS, GHQ-28, MBI-GS, UWES-9, and Minnesota Satisfaction Questionnaire (101–103). Across 28 European National Nurses’ Associations, psychological, physical, and sexual violence was confirmed in 18 countries, with sexual harassment up to 30% and abuse from colleagues up to 41%, resulting in trauma, stress, burnout, emotional exhaustion, humiliation, low job satisfaction, reduced working hours, and turnover intentions (104).

Overall, European research illustrates a trade-off between contextual richness and standardization: Switzerland and Germany prioritize validated instruments for reliable prevalence estimates, whereas Belgium, France, Spain, and Italy employ bespoke or mixed-method approaches to capture subtle aggression, internal sources, and workplace dynamics (95–104). Italy offers the most comprehensive but heterogeneous evidence base. Despite extensive descriptive data, empirical evaluation of preventive interventions (i.e., training, policy implementation, or measurable outcomes) remains largely absent or only suggested, highlighting a critical gap in translating WPV epidemiology into actionable workplace safety strategies (86–94).

#### 3.3.4 America and Australia

Recent research from North and South America and Oceania (*n* = 12) highlights the high prevalence of WPV and its impact across professional roles and clinical settings (Table 3). HCWs are predominantly female, particularly among nurses, home care workers, and oncology and surgical specialists (105–107). Differences between high-income, war-free systems (United States, Canada, Australia) and middle-income contexts with social instability (Colombia, Mexico, Peru) suggest that institutional resources and socio-political conditions strongly influence WPV risk, reporting, and outcomes (108).

In North America, WPV affects ED staff, surgical residents, physicians, and home care workers, with high-risk settings including emergency departments, psychiatric units, and academic hospitals. Psychological violence (including verbal abuse, threats, bullying, harassment, gender-based exclusion) is most frequent (about 70%) (105, 109). Physical violence occurs mainly in high-acuity EDs and psychiatric settings, and sexual harassment is common, particularly among female HCWs, involving both external (patients, visitors) and internal (supervisors, colleagues) perpetrators. Underreporting remains substantial; ED staff reported verbal abuse increasing from 6% pre-pandemic to 13% during the pandemic, and sexual harassment prevalence reached 60% among ophthalmology trainees, although only 24% of cases were reported (105, 109, 110).

WPV exposure and consequences were assessed using validated and study-specific instruments, including the Maslach Burnout Inventory, MHI-5, SF-36, Oldenburg Burnout Inventory, Veterans Affairs All Employee Survey, NIOSH Worker Well-Being Questionnaire, and the Negative Acts Questionnaire (106, 111–113). Consequences included burnout, depression, PTSD, impaired work-life balance, turnover intentions up to 70%, reduced job satisfaction, and chronic health issues. Internal harassment was also linked to imposter syndrome and limited career advancement (113, 114). Intervention evidence was limited, although a Massachusetts ED study demonstrated that a team-based agitation management protocol reduced physical assaults and restraint use (115).

In Latin America, studies focused on oncologists in Mexico, Chile, Argentina, and Peru, and surgical residents in Colombia. Psychological violence and sexual harassment were common, with both internal and external perpetrators contributing. Female HCWs were disproportionately affected, experiencing impacts on career progression, income, and mental health, including burnout, depression, and PTSD (107, 116). Assessments relied primarily on the Negative Acts Questionnaire, self-reported harassment measures, and the ESMO Women for Oncology survey, with no intervention studies reported (107, 116).

Australian research examined nurses in mental health units, where verbal and physical aggression primarily from patients affected safety and psychological well-being (117). The randomized trial showed that combining eDASA with an Aggression Prevention Protocol reduced aggressive incidents, verbal abuse, psychotropic medication use, and seclusion episodes while increasing de-escalation and early intervention (117).

Overall, WPV across the Americas and Oceania is widespread, with psychological violence predominating. Contextual factors influence reporting and mitigation, with high-income countries showing greater capacity for intervention. Evidence on prevention remains limited, and apart from the Australian trial, most studies are descriptive, underscoring the need for context-specific, multi-level approaches (17, 34).

#### 3.3.5 Global evidence

Only two global cross-sectional studies were identified, highlighting limited internationally comparable evidence (Table 3). A survey among cardiologists found that 44% experienced a hostile work environment, including emotional harassment (29%), discrimination (30%), and sexual harassment (4%), with higher prevalence among women (118). Perpetrators included internal actors (administrative staff, colleagues, senior physicians) and patients. Exposure was associated with anxiety, fear, and reduced professional satisfaction (118).

A study on renal nurses across Europe, the Middle East, and Australia reported that 73% had observed at least one violent incident over three years, mainly psychological aggression from patients or caregivers, linked to burnout and reduced job satisfaction (119). Both studies lacked intervention components and noted substantial underreporting, with incidents documented mainly when severe, highlighting gaps on global evidence (118, 119).

## 4 Discussion

This scoping review confirms that psychological and verbal violence are the most prevalent forms of WPV globally (Table 3). Recent evidence indicates that prevalence has increased over the past five years, particularly in emergency and psychiatric settings, highlighting a growing occupational health concern amplified by pandemic-related stressors (Table 1). Physical and sexual violence, although less frequent, remain clinically significant in high-risk settings such as EDs, psychiatric wards, and long-term care facilities (51, 105). Regional prevalence varies, with Europe reporting 10–76% physical violence, Africa showing psychological and verbal violence exceeding 50% in several countries, and Asia exhibiting predominance of verbal and psychological aggression, mostly from patients and relatives (44, 45, 56, 63, 86, 100).

Nurses, early-career professionals, and other staff (physicians, midwives, allied health, and administrative personnel) are at heightened risk due to patient-facing roles and limited experience (28, 29, 67, 71). Gender disparities are evident, with female healthcare workers reporting higher rates of psychological, physical, and sexual violence (96, 109). WPV arises from both external perpetrators (patients, relatives) and internal sources (colleagues, supervisors). Underreporting remains pervasive, driven by fear of retaliation, perceived ineffectiveness of reporting systems, and normalization of aggression, highlighting the need for transparent, culturally sensitive reporting mechanisms (83, 86, 93, 96).

Determinants of WPV are multifactorial, spanning individual, organizational, and contextual levels. High-risk units are characterized by high patient acuity, workload, and emotional demand (30, 51). Organizational contributors include staffing shortages, workload, and weak institutional policies (75, 118). Low- and middle-income and conflict settings show higher risk and lower reporting, while high-income countries remain affected by organizational factors (40, 100).

In addition to routine workplace violence, recent reports have highlighted the escalation of targeted attacks against healthcare workers and healthcare facilities in conflict-affected and war-zone settings (120, 121). These extreme forms of violence represent a critical extension of WPV, further exacerbating occupational risks, disrupting service delivery, and limiting both reporting and protective mechanisms (122). Such contexts underscore the importance of situating workplace violence within broader structural and geopolitical determinants of health system safety (123, 124).

WPV is associated with psychological effects (burnout, anxiety, depression, PTSD), professional impacts (reduced job satisfaction, absenteeism, turnover), and compromised patient care, highlighting the interdependence of occupational safety, workforce retention, and clinical quality (44, 49, 53, 69, 71, 75, 96, 102).

Cross-continental differences in WPV patterns reflect variations in health system capacity, workforce availability, cultural norms, and reporting practices. High-income regions tend to show more structured reporting systems and greater availability of institutional interventions, although underreporting remains substantial (95, 99, 106, 115). In contrast, low- and middle-income and conflict-affected settings often experience higher exposure combined with weaker reporting infrastructures and limited preventive resources (40, 47, 58, 68). These disparities suggest that both the observed prevalence and the effectiveness of mitigation strategies are strongly context-dependent.

Preventive strategies include organizational measures (policies, reporting systems, environmental safety, training) and psychosocial support (through counseling and peer networks) (70, 115, 117). Individual-focused interventions, including situational prevention programs, interactive educational tools, and adaptive coping techniques, may improve coping and resilience (125, 126). System-level frameworks (such as SEIPS 3.0-based Canadian initiatives), culturally tailored coping policies (e.g., Iranian nurses’ “active endurance”), and legislative interventions (Turkey’s 2022 healthcare violence law), highlight the importance of context-sensitive, multilevel strategies, although implementation remains inconsistent (80, 127–129).

Importantly, the effectiveness, availability, and implementation of preventive strategies also vary across geographical and health system contexts (37, 72, 104, 108). High-income settings more frequently report structured institutional frameworks and integrated prevention models (104, 108). Whereas in low- and middle-income countries strategies are often constrained by resource limitations, weaker enforcement of policies, and underdeveloped occupational safety systems (37, 40). These disparities reflect broader global health system inequalities and governance differences influencing workplace violence prevention capacity (35, 36). Globally, legislative protections, including national laws and international frameworks such as ILO Convention No. 190, are inconsistently applied, highlighting the need for harmonized policy adoption and enforcement (130).

Ongoing studies are leveraging innovative data collection methods (e.g., digital surveys, biofeedback, chatbot reporting) to address underreporting and context-dependent violence (72, 131) (Supplementary Table S3). Recent evidence from Europe, Asia, and North America continues to confirm the high prevalence and adverse consequences of WPV among HCWs, while highlighting the importance of standardized policies, staff debriefing, psychosocial support, systematic training, and multilevel prevention strategies (132–142). A recent narrative review from Asia further corroborates these findings, highlighting similar patterns of WPV against HCWs and reinforcing the need for context-specific prevention strategies (143). Finally, AI-based approaches, including predictive modeling, natural language processing, and immersive training tools, are emerging as promising strategies to strengthen multilevel WPV prevention by enabling early risk detection, improving patient–provider communication, and enhancing workforce preparedness across healthcare settings (144–147).

Limitations of this review include the absence of formal quality appraisal, predominance of cross-sectional self-report studies, heterogeneity in definitions and tools, and geographic skew toward high-income countries. Additionally, the absence of formal protocol registration may be considered a minor limitation; however, the review followed established methodological guidance to ensure transparency and methodological rigor (21, 26). These factors may limit generalizability, particularly to low-resource or conflict-affected settings; while the exclusion of non-English studies may introduce selection bias. Future research should prioritize longitudinal and intervention studies with standardized, context-sensitive tools to generate actionable evidence for WPV prevention.

## 5 Conclusion

Workplace violence represents a universal, multidimensional challenge affecting healthcare workers worldwide, with nurses, women, and early-career professionals at highest risk. External (patients, relatives) and internal (colleagues, supervisors) perpetrators create a dual-risk environment, underscoring the need for integrated, context-sensitive prevention combining organizational policies, environmental design, staff training, and psychosocial support.

Advancing the evidence base requires robust, standardized, and intervention-focused research, particularly in low-resource and conflict-affected settings, to develop scalable and sustainable solutions. The integration of digital and AI-based tools offers additional opportunities to enhance early detection, communication, and workforce preparedness, provided that implementation remains ethical, equitable, and context-sensitive.

Ultimately, multilevel approaches addressing organizational and systemic determinants are essential to reduce WPV, improve reporting, and mitigate its psychological, occupational, and social impacts.
