# Supplementary material for: The rising tide of workplace violence in the healthcare sector: a global scoping review of burden, determinants, and prevention strategies (2021–2025)
Source: Front Public Health. 2026 May 14;14:1851734. doi: 10.3389/fpubh.2026.1851734 (PMC13216234; doi:10.3389/fpubh.2026.1851734)
Supplement: Supplementary file 1 [file Data_Sheet_1.docx]

Supplementary Material

# Supplementary Figures and Tables

## Supplementary Tables

**Supplementary Table S1. Summary of free-text keywords used in the search**

| **Conceptual domain** | **Keywords** |
| --- | --- |
| Workplace violence and harassment | workplace violence; violence; harassment; bullying; mobbing; sexual harassment; verbal abuse; physical violence; psychological violence; aggression |
| Healthcare personnel | healthcare workers; healthcare personnel; health personnel; nurses; physicians; doctors; medical staff; allied health professionals; paramedics; residents; medical residents; interns; healthcare students; nursing students |
| Healthcare settings | hospital; healthcare setting; emergency department; emergency room; psychiatric unit; mental health services; outpatient clinic; long-term care; nursing home; primary care |

**Supplementary Table S2. Medical Subject Headings (MeSH) used in the search strategy, with selected entry terms, scope notes, and MeSH unique identifiers**

| **MeSH heading** | **Selected entry terms (examples)** | **Scope note (abridged MeSH definition)** | **MeSH UI** |
| --- | --- | --- | --- |
| **Workplace Violence** | Occupational violence; Work-related violence | Physical or psychological harm occurring in occupational settings, including threats, abuse, or assaults directed at workers. | D064450 |
| **Violence** | Interpersonal violence; Physical violence | Use of physical force or power, threatened or actual, resulting in injury, harm, or psychological damage. | D014754 |
| **Sexual Harassment** | Unwanted sexual attention; Sexual intimidation | Unwelcome sexual advances or conduct of a sexual nature occurring in employment or educational settings. | D017406 |
| **Harassment, Non-Sexual** | Non-sexual harassment; Irritating actions; threats | Act of systematic and/or continuous unwanted and irritating actions of a non-sexual nature (include bullying etc.). | D000071277 |
| **Exposure to Violence** | Violence exposure; Experience of violence | Experience of and exposure to violence. | D000069581 |
| **Bullying** | Workplace bullying; Mobbing | Repeated aggressive behavior involving power imbalance and resulting in harm or distress. | D058445 |
| **Aggression** | Hostile behavior; Violent behavior | Behavior intended to harm or intimidate another individual physically or psychologically. | D000374 |
| **Health Personnel** | Healthcare workers; Medical staff | Individuals working in healthcare delivery, including professionals, trainees, and support staff. | D006282 |
| **Nurses** | Registered nurses; Nursing staff | Individuals trained and licensed to provide nursing care. | D009726 |
| **Physicians** | Medical doctors; Clinicians | Individuals licensed to practice medicine. | D010820 |
| **Students, Health Occupations** | Medical students; Nursing students | Individuals enrolled in education programs for healthcare professions. | D013336 |
| **Internship and Residency** | Medical residency; Clinical training | Structured postgraduate training programs in healthcare professions. | D007396 |
| **Hospitals** | General hospitals; Teaching hospitals | Institutions providing medical and surgical treatment and nursing care. | D006761 |
| **Emergency Service, Hospital** | Emergency department; Emergency room | Hospital units providing immediate care for acute illness or injury. | D004636 |
| **Psychiatry** | Psychiatric services | Medical specialty focused on mental disorders and their treatment. | D011570 |
| **Mental Health Services** | Behavioral health services | Organized services delivering mental health care. | D008605 |
| **Long-Term Care** | Nursing homes; Residential care | Health and personal care services provided over extended periods. | D008134 |

**Supplementary Table S3. Ongoing and completed clinical studies on workplace violence in healthcare**

| **NCT Number** | **Title** | **Recruitment Status** | **Study Type** | **Study Population** | **Eligibility** | **Healthcare Setting** | **How Violence is Studied** | **Intervention** | **Publication(s)** |
| --- | --- | --- | --- | --- | --- | --- | --- | --- | --- |
| NCT05419687 | Violence Against Health Care Workers in Fragile Settings (VIA-H) | Completed | Interventional; stepped-wedge cluster-randomized trial; crossover assignment; double masking (investigator and outcome assessor); health services research | HCWs: nurses in DRC; junior doctors and permanent staff; N = 798 | Inclusion: HCWs ≥6 months employment, consented; Exclusion: age <18, cognitive impairment | Rural health facilities in DRC (Bukavu); secondary hospitals in Baghdad, Iraq | Self-reported: verbal, physical aggression; PTSD, burnout, empathy, coping, absenteeism, turnover intention | De-escalation training + code of conduct (citizen science approach); 11 intervention sequences | (1) |
| NCT05796323 | Perceptions and Prevalence of Workplace Violence in Healthcare | Enrolling by invitation | Observational; prospective; ecologic/community model; non-probability sample | Employees of Methodist Health System (clinical and non-clinical), estimated N = 3000 | Inclusion: full-time, part-time, PRN employees; Exclusion: inability to read English | Acute care hospitals, main site: Methodist Dallas Medical Center, Dallas, TX, USA | Self-reported survey via SurveyMonkey; type/level/frequency of violence, impact on workers and system | None | None reported |
| NCT06921083 | Violence in ICU: Outcomes, Lessons, and Experiences of ICU Team (The VIOLENT-Study) | Recruiting | Observational; prospective; probability sample | Nurses and physicians in ICUs; estimated N = 500 | Inclusion: consenting nurses and physicians; Exclusion: non-consenting | ICUs in Switzerland, main site: University Hospital Basel | Questionnaire: frequency, type, triggers, reporting, personal/professional consequences, prevention strategies | None | None reported |
| NCT06129929 | Effectiveness of Using Jiu-Jitsu for Coping With Medical Violence in HCWs | Completed | Interventional; non-randomized; parallel assignment; single masking; prevention purpose | Licensed HCWs (mainly nurses); N = 396 | Inclusion: licensed HCWs; Exclusion: non-licensed | Hospital, Taiwan (TSGH, Taipei) | Validated scales: self-efficacy, perception of aggression, attitudes, turnover intention; pre-post | Behavioral: Brazilian jiu-jitsu self-defense training vs. traditional lecture | (2) |
| NCT07473531 | Chatbot to Support Healthcare Professionals Experiencing Workplace Aggression | Not yet recruiting | Observational; prospective; case-only; non-probability; mixed-methods | HCWs in emergency/critical care; N = 223 | Inclusion: active clinical practice, ≥18, consent; Exclusion: undergoing formal intervention, refusal | Emergency and Critical Care Units, Almería Health District, Spain | Interaction with chatbot; usability (SUS), readability (INFLESZ), qualitative feedback | Digital: SANIDAD SEGURA chatbot providing guidance, coping strategies, legal/institutional support | None reported |
| NCT04492085 | National Survey of Workplace Violence | Recruiting | Observational; prospective cohort; probability sampling | Obstetrics/Gynecology physicians; N = 6000 | Inclusion: ≥18, active employment, informed consent; Exclusion: incomplete or ineligible | Multi-center across mainland China (primary, secondary, tertiary hospitals) | Questionnaire via WeChat; prevalence of verbal, physical, sexual violence; causes and solutions | None | None reported |
| NCT04115332 | Effects of Resilience and Mental Health of Abused Psychiatric Nurses: Biofeedback Training | Completed | Interventional; quasi-experimental; supportive care; open-label | Abused psychiatric nurses; N = 136 | Inclusion: psychiatric ward nurses exposed to violence; Exclusion: not specified | Psychiatric setting; Kaohsiung Medical University Hospital, Taiwan | Psychological (CES-D, resilience, occupational stress) and physiological (EEG, HRV, skin conductance) pre-post | Biofeedback training; comparison: video relaxation, no intervention | None reported |
| NCT05484986 | Nursing Students' Personality Traits and Competence in Workplace Violence Management | Completed | Observational; cross-sectional | Nursing students; N = 321 | Inclusion: age >18, consent; Exclusion: age <18, refusal | Nursing education; Bursa Uludag University, Turkey | Questionnaire: self-reported violence exposure, competence (MWVCS), personality (BFI) | None | Not provided |
| NCT07311629 | Workplace Violence and Safety Perception Among ENT Physicians in Turkey | Not yet recruiting | Observational; Patient Registry; cross-sectional | ENT-HNS specialist/resident physicians ≥18, N = 450 | Inclusion: licensed ENT-HNS, consent; Exclusion: other specialties, surveys >20% missing | Multicenter ENT clinical practice, Turkey | Anonymous online survey; exposure to verbal, physical, threats; perceived safety (0–10 Likert) | None | Not provided |
| NCT06933433 | Assaults Against Health Care Professionals in a Tertiary ICU | Not yet recruiting | Observational; retrospective cohort | Adult patients (≥18) who committed ICU violence against HCWs; N = 865 | Inclusion: ≥18, reported ICU incident; Exclusion: none | Single-center tertiary ICU, University Hospital Basel, Switzerland | Retrospective digital medical record review (2011–2024) for incident type, context, consequences | None | Not provided |

**References:**

1. Ferrari G, Lwamushi SM, Balaluka GB, Lafta RK, Schindler C, Bugugu D, Lurhangire E, Tediosi F, Mendoza JR, Merten S. Understanding context of violence against healthcare through citizen science and evaluating the effectiveness of a co-designed code of conduct and of a tailored de-escalation of violence training in Eastern Democratic Republic of Congo and Iraq: a study p. *Trials* (2023) 24:814. doi: 10.1186/s13063-023-07839-3

2. Ma C-Y, Liao S-J, Chang Y-C, Chiang H-H. Effectiveness of a theory-driven Brazilian jiu-jitsu-based medical self-defense training for nurses facing workplace violence: A multicenter quasi-experimental study. *Int J Nurs Stud* (2026) 173:105260. doi: 10.1016/j.ijnurstu.2025.105260

## Supplementary Figures


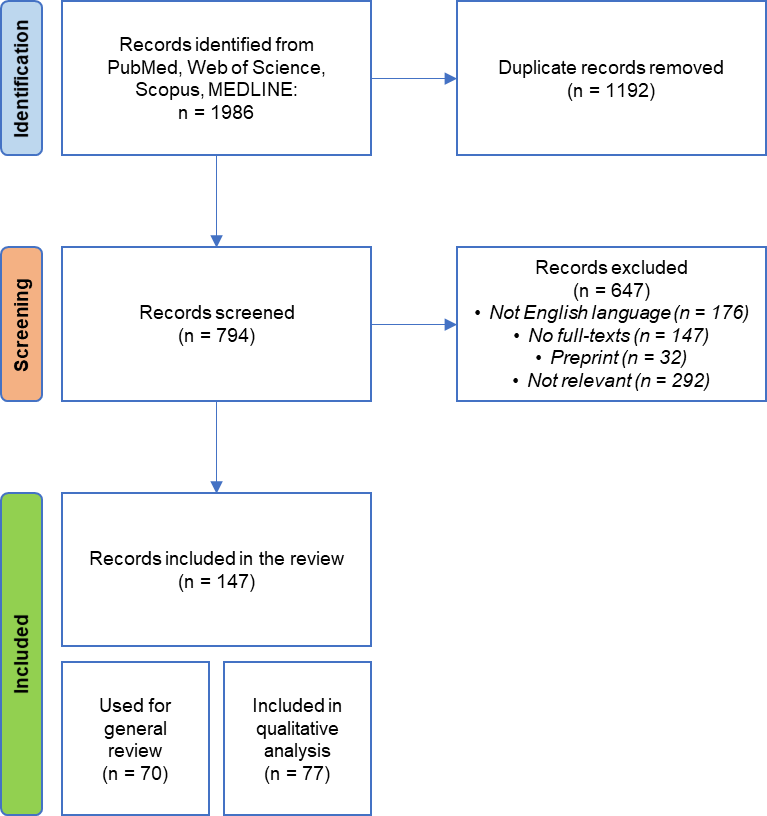


**Supplementary Figure 1. PRISMA flow diagram of study selection process.** The figure illustrates the study identification, screening, eligibility, and inclusion phases of the scoping review. Records identified through database searching and other sources were first screened for duplicates and relevance. A total of 147 records were included in the review, of which 77 were retained for qualitative analysis.
